# Exploring the feasibility and acceptability of DIALOG+ (a structured digital communication tool) in strengthening psychiatric care in India and Pakistan: a qualitative pilot study

**DOI:** 10.1136/bmjopen-2024-091852

**Published:** 2025-08-12

**Authors:** Onaiza Qureshi, Kasthuri Divya, Mehwish Dawood, Syjo Davis, Lakshmi Venkatraman, Maria Baig, Krishna Priya, Renata Peppl, Manikandan Pari, Padmavati Ramachandran, Aneeta Pasha, Sana Zehra Sajun, Hufsa Sarwar, Areeba Shahab, Victoria Jane Bird

**Affiliations:** 1 Interactive Research & Development, Karachi, Pakistan; 2 Schizophrenia Research Foundation, Chennai, Tamil Nadu, India; 3 Unit for Social and Community Psychiatry, Queen Mary University of London, London, UK

**Keywords:** PSYCHIATRY, QUALITATIVE RESEARCH, Schizophrenia & psychotic disorders, Psychosocial Intervention, Patient-Centered Care

## Abstract

**Abstract:**

**Objectives:**

To assess the implementation feasibility and acceptability of a structured digital psychosocial communication tool (DIALOG+) to strengthen the quality of person-centric care in psychiatric settings within Pakistan and India.

**Design:**

A hybrid inductive and thematic qualitative analysis using individual interviews (IDIs) and focus group discussions (FGDs).

**Setting:**

Two psychiatric hospitals (Karwan-e-Hayat and Jinnah Postgraduate Medical Centre) in Karachi, Pakistan and one psychiatric care organisation (Schizophrenia Research Foundation) in Chennai, India

**Participants:**

Interviews were conducted with 8 mental health clinicians and 40 patients who completed the DIALOG+ pilot as well as wider stakeholders, that is, 12 mental health clinical providers, 15 caregivers of people with psychosis and 13 mental health experts.

**Intervention:**

A technology-assisted communication tool (DIALOG+) to structure routine meetings and inform care planning, consisting of monthly sessions over a period of 3 months. The intervention comprises a self-reported assessment of patient satisfaction and quality of life on eight holistic life domains and three treatment domains, followed by a four-step solution-focused approach to address the concerns raised in chosen domains for help.

**Outcome measures:**

Key insights for the implementation feasibility and acceptability of DIALOG+ were assessed qualitatively using inductive thematic analysis of 22 IDIs and 8 FGDs with 54 individuals.

**Results:**

Clinicians and patients ascribed value to the efficiency and structure that DIALOG+ introduced to consultations but agreed it was challenging to adopt in busy outpatient settings. Appointment systems and selective criteria for who is offered DIALOG+ were recommended to better manage workload. Caregiver involvement in DIALOG+ delivery was strongly emphasised by family members, along with pictorial representation and relevant life domains by patients to enhance the acceptability of the DIALOG+ approach.

**Conclusion:**

Findings highlight that the feasibility of implementing DIALOG+ in psychiatric care is closely tied to strategies that address clinician workload. Promoting institutional ownership in strengthening resource allocation is essential to reduce the burden on mental health professionals in order to enable them to provide more patient-centric and holistic care for people with psychosis. Further research is required to explore the appropriateness of including caregivers in DIALOG+ delivery to adapt to communal cultural attitudes in South Asia.

**STRENGTHS AND LIMITATIONS OF THIS STUDY**

- The study recruited both active participants to DIALOG+ as well as a wider pool of caregivers of people with psychosis, mental health clinicians and experts in both countries to include more diverse and contextual input into the feasibility and accessibility of DIALOG+.
- Conducting both in-person and online interviews improved accessibility for participants who were unable to travel to clinical sites, though poor internet connection sometimes hindered engagement in the online modality.
- Due to the gap of about a month between the delivery of DIALOG+ sessions and the subsequent interviews, some participants had difficulty accurately recalling their experiences, potentially introducing recall bias into the responses.
- The researchers conducting the interviews were also involved in recruiting and coordinating with the participants, which may have introduced the potential for desirability bias.

## Introduction

 Mental, neurological and substance use disorders significantly contribute to the global burden of disease and are the leading cause of years lived with disability.^1^ Psychotic disorders such as schizophrenia and mood disorders with co-occurring psychotic features such as bipolar disorder are among the most severely disabling of mental health conditions.^2 3^ They often present during teenage or early adulthood years^4^ and can become long-term conditions that are associated with significant rates of premature mortality (relative to the general population),^5 6^ social isolation and exclusion,^7^ socioeconomic disadvantage^8^ and human rights abuses.^9^ Further, people living with psychotic disorders tend to have a higher treatment gap of up to 96% (referring to the gap between the proportion of people who require care but do not receive it), a longer duration of untreated illness, receive lower quality of healthcare and are less likely to adhere to medication plans leading to poorer health and social outcomes.^10^

Global literature suggests that these adverse outcomes are often aggravated in vulnerable groups living in low- and middle-income countries (LMICs) like Pakistan and India, where the mental health workforce is under-resourced to address the psychiatric need.^11^ With fewer than 400 psychiatrists in Pakistan and 0.75 psychiatrists for 100 000 people in India, many of whom are clustered in urban settings, care for people living with psychotic disorders is largely isolated in tertiary care facilities, biomedical in nature (eg, prioritising pharmacological solutions for mental health concerns) and limited in capacity for psychosocial interventions despite global evidence for their effectiveness in facilitating recovery.^12^,^14^ While India provides mental healthcare through specialist, district and teaching hospitals as part of their National Mental Health Programme, little is known about the treatment of psychotic disorders across varied mental health facilities in Pakistan.^15^

Multidisciplinary care involving a complex package of pharmacotherapy, treatment plan management, psychosocial therapy, family intervention and follow-up care through community outreach is globally recommended for maintenance treatment to reduce symptomatology, need for inpatient stays, enhance quality of life and improve interpersonal skills.^16^ However, due to a high patient flow volume and limited training opportunities, mental health practitioners in Pakistan and India feel unable to provide holistic care, despite having the capacity and interest to do so.^17 18^ These challenges underscore the need to explore and evaluate the contextual feasibility and cultural appropriateness of resource-effective and patient-centred approaches to enhance care in these settings.

An evidence-based and technology-assisted approach called DIALOG+ is one low-cost solution that has shown efficacy in improving the quality of mental health consultations with minimal training and institutional resources required.^19^ DIALOG+ uses a person-centred solution-focused model and has been tested across a range of high and low-resource settings and mental health conditions, where it has been shown to improve quality of life and treatment outcomes for people with long-term treatment needs.^20^,^22^ The approach involves a patient-centred assessment on holistic life and treatment domains followed by a four-step solution-focused approach to address patient concerns. Clinicians can use it on an application available on tablets or phones within routine clinical meetings.^20^

The National Institute for Health Research-funded project ‘Improving outcomes for people with psychosis in Pakistan and India – enhancing the Effectiveness of Community-based care (PIECEs)’ was launched in September 2020, to enhance the quality of psychiatric care in Indian and Pakistan using the DIALOG+ approach. Initially, DIALOG+ was piloted over 3 months in psychiatric settings within Karachi, Pakistan and Chennai, India to assess the feasibility and acceptability of the approach and inform subsequent cultural adaptations to South Asian mental healthcare systems.^23 24^ This paper reviews the perspectives and experiences of pilot participants as well as clinicians, caregivers and local mental health experts to achieve the following aims:

1. To qualitatively assess the feasibility of delivering DIALOG+ in mental health clinical settings.
2. To understand perceptions around the acceptability of DIALOG+ for beneficiaries (service users, mental health clinicians and caregivers) in Pakistan and India.

## Methods

### Study design

This is a qualitative study investigating the feasibility and acceptability of delivering DIALOG+ which is used to enhance structure and person-centred care principles in clinical consultations. The study sample included patients with psychosis (40) and mental health clinicians (8) enrolled in the DIALOG+ pilot within the PIECEs research programme, as well as naive individuals (defined as participants who had no prior experience of DIALOG+) including mental health clinicians (12) who had not been introduced to DIALOG+, caregivers of people with psychosis (15) and local mental health experts (13).

### Setting

The setting for the DIALOG+ pilot study took place at two mental health clinical settings in Karachi, Pakistan and one mental health clinical centre in Chennai, India.

- Karwan-e-Hayat (KEH) is a charity-run mental health institute that offers a range of subsidised mental health inpatient and outpatient services for persons with psychiatric illnesses.
- The Department of Psychology and Behavioural Sciences at Jinnah Postgraduate Medical Centre (JPMC) is a public tertiary mental health facility where all mental health services and medication are offered free of cost.
- The Schizophrenia Research Foundation (SCARF) is a non-governmental organisation that provides mental healthcare services including rehabilitation of persons with severe mental health conditions.

DIALOG+ sessions took place in outpatient consultation rooms in SCARF in India, in psychiatric consultation rooms within the Rehabilitation department at KEH and the Schizophrenia Outpatient Department (OPD) at JPMC in Pakistan.

Each DIALOG+ session begins with the patient and their clinician using the tablet to rate their satisfaction on eight life domains (mental health, physical health, job situation, accommodation, leisure activities, friendships, relationships with family/partner and personal safety) and three treatment domains (medication, practical help and meeting with professionals). Each satisfaction item is rated on a scale from 1 (‘totally dissatisfied’) to 7 (‘totally satisfied’) and followed by a question on whether the patient wants additional help with that domain. Ratings are followed by a four-step solution-focused approach to address concerns raised within each domain selected for additional help. The four steps taken by clinicians and patients are: (1) understanding, (2) looking forward, (3) exploring options and (4) agreeing on actions.^19^

### Sample selection

Participants were recruited between 17 August 2021 and 25 September 2021, with two purposively selected samples to enhance the diversity of perspectives on DIALOG+ feasibility and acceptability within local settings:

1. Individuals who took part in the DIALOG+ pilot:
  - 8 mental health clinicians (4 in India and 4 in Pakistan) and 40 people with psychosis (20 in India and 20 in Pakistan) who took part in the DIALOG+ pilot.
2. Individuals who had no prior experience of DIALOG+
  - 15 caregivers of enrolled participants (7 in India and 8 in Pakistan), 16 naïve mental health professionals (eight from India and Pakistan each) who had never used DIALOG+ and a mixed stakeholder group of 15 mental health and industry experts (8 in India and 5 in Pakistan) from service delivery or advocacy organisations were invited to take part in the interviews for their perspectives on the approach. Since the overarching project aimed to scale up DIALOG+ implementation across additional mental health settings and clinicians, including these stakeholders was necessary in order to understand feasibility and acceptability perspectives into the broader mental health infrastructure, resources and context that would influence the adoption of DIALOG+. Prior to the interviews, these individuals were given an orientation to DIALOG+ protocol and clinical application so that they were familiar with its intended purpose and would be better able to respond to the questions.

Individuals with psychosis were considered eligible if they took part in the 3-month DIALOG+ pilot, spoke the local language, were between 18 and 65 years, had a clinical diagnosis of schizophrenia, schizotypal, delusional and other non-mood psychotic disorders (as per the tenth revision of the International Classification of Diseases (ICD-10) codes F20–29), or bipolar disorder with psychotic features (F31.2, F31.5, F31.64), were capable of providing informed consent, were not admitted for psychiatric care and had an illness duration of more than 2 years. Mental health clinicians were eligible if they were aged 18 years or above, currently employed in a mental healthcare role, had prior experience of working with individuals with psychosis and spoke the local language. Clinicians with and without prior experience of DIALOG+ were recruited into the study. Family members or carers of individuals with psychosis were eligible if they were 18 years or older and spoke the local language. Lastly, the study included mental health experts who are actively engaged in clinical services and had experience of working with individuals suffering from psychosis.

### Data instruments

A semistructured interview guide was developed for use in both in-depth interviews (IDIs) and focus group discussions (FGDs) (online supplemental file 1). It included questions about (1) the experiences, perceived barriers and facilitators of DIALOG+ by participants, (2) suggestions on the approach, for example, visuals, font, content, domains, materials and (3) practical delivery of the approach for informing cultural adaptations. This guide was modified from an existing version used for evaluating DIALOG+ in similar low-income settings.^25^

A sociodemographics form was developed and administered to participants to understand the basic participant characteristics including age, gender, language and ethnicity. The form also included specific questions for caregivers, that is, relationship with and duration of caring for the person with psychosis for caregivers and for clinicians, that is, years of experience working as a mental health clinician and professional background.

### Study procedures (DIALOG+)

The DIALOG+ pilot was conducted for 3 months between 2 June 2021 and 30 August 2021 in Pakistan and 5 May 2021 to 16 October 2021 in India. It involved clinicians using the DIALOG+ approach with patients attending routine consultations at each clinical site. After the pilot, we contacted clinicians and participants to invite them for a face-to-face IDIs or FGDs to discuss their experiences with DIALOG+. Additionally, caregivers, naïve clinicians and mental health experts were identified from SCARF and Interactive Research & Development (IRD)’s personal networks, assessed as per the eligibility criteria and invited to take part in face-to-face FGDs to provide their perspectives on the DIALOG+ approach. They were contacted by phone or email to attend a meeting with researchers (OQ, AS, SD, LV) who had received training on qualitative data collection methods. Among the researchers involved in recruiting and interviewing, OQ and LV were co-investigators of the project. OQ, AS and LV are women, and SD is a man. At the time of the study, LV was a psychiatrist (MRCPSYCH), OQ was a programme manager (MSc in Global Mental Health), AS was a research associate (MSc in Psychology) and SD was a programme manager (MS in Social Work).

Participants already knew about the researchers and their role in this research as part of a wider pilot for DIALOG+ at each of the participating sites. Naïve participants were introduced to the project after the pilot and were informed of the researchers’ intentions to better understand the feasibility and acceptability of DIALOG+ with the aim to scale up the interventions across additional clinicians at the participating psychiatric institutions. Bias was managed by ensuring that none of the researchers were involved in the clinical management of the participants receiving care at the institutions where they were recruited for the interview.

IDIs and FGDs were facilitated by two researchers, ranged between 35 and 90 min and were conducted either in-person at clinical sites or virtually via Zoom or phone call, depending on the participants’ availability. For participants unfamiliar with the DIALOG+ approach, the FGDs (containing between 5 and 8 individuals) began with an introduction to DIALOG+, followed by ground rules to facilitate the group discussion and emphasise the importance of privacy and confidentiality (online supplemental file 1). All interviews and group discussions were audiorecorded using recorders and uploaded to a secure drive. Recordings were transcribed (verbatim), translated and anonymised by SCARF and IRD researchers, with supplementary notes taken during the sessions. Transcripts were imported into NVIVO 12.

### Data analysis

As the primary focus of the study was to assess the feasibility and acceptability of DIALOG+, the qualitative analysis undertook an inductive approach to code and analyse the IDIs and FGDs using elements of thematic analysis for the synthesis. This hybrid approach to analysis combined inductive thematic analysis with directed elements which has been showcased by Fereday and Muir-Cochrane^26^ to allow thematisation to emerge organically while still allowing researchers to incorporate relevant programmatic objectives (ie, feasibility and acceptability) when exploring participant inputs.^26^ Moreover, data saturation was not a primary consideration in this qualitative analysis as our focus at this stage was to evaluate the feasibility and acceptability of DIALOG+ within the broader research project, with the goal of informing subsequent adaptations to optimise its adoption during wider implementation among psychiatric clinicians.

**Figure 1:**
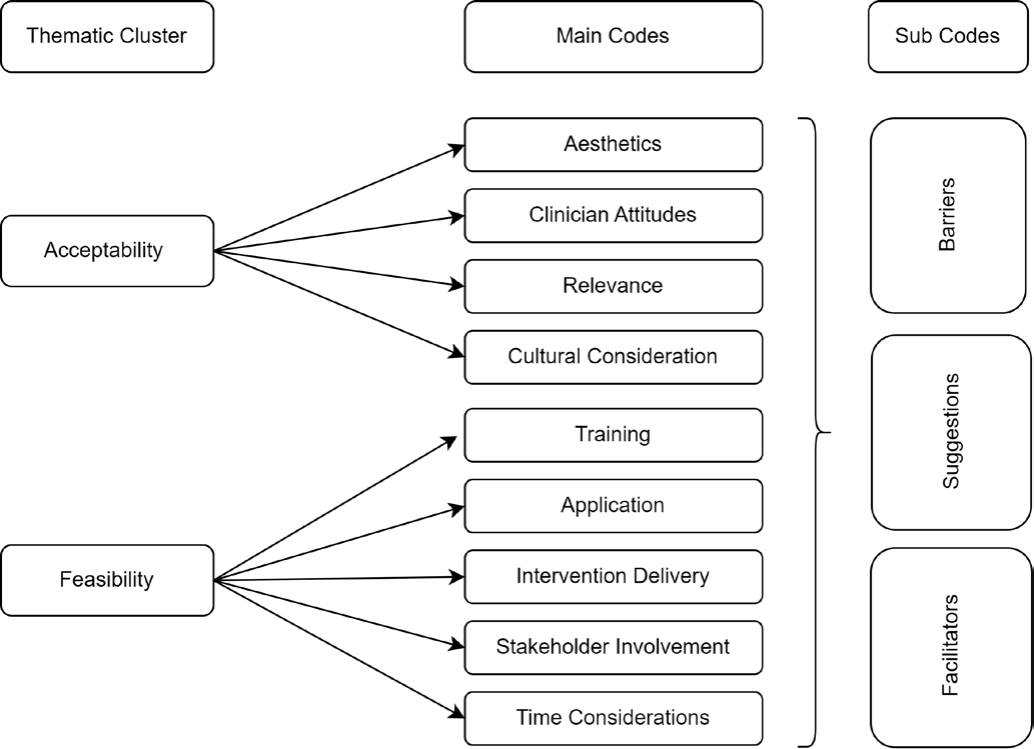
Coding framework for thematic clusters, parent codes and subcodes.

A two-stage process was used, wherein four researchers from SCARF (SD, KD, LV and PR) and IRD (OQ, MD, AS and HS) each familiarised themselves with two transcripts from India and Pakistan each and conducted open coding to develop and apply codes for emerging topics under the two main themes of feasibility and acceptability. Care was taken to critically reflect on the codes, to ensure unbiased definitions were arrived at, employing the strategy of reflexivity and continually questioning and reflecting on decisions. Meetings were conducted between the research team to establish consensus for the merging of the resulting codes into a consolidated codebook (figure 1) where any disagreements were discussed and addressed with appropriate justification and finalised by the primary investigator VJB. In the second stage of the analysis, coding was conducted on all interview transcripts by researchers from IRD and SCARF. Findings were extracted and consolidated in one NVIVO file for subsequent analysis of the results.

### Patient and public involvement

Drafts of the IDI and FGD scripts were shared with members of the PIECEs Lived Experience Advisory Panel for review before finalising; however, no revisions were suggested or made by the group.

## Results

In total, 9 FGDs (5 from Pakistan and 4 from India) and 22 IDIs (9 from Pakistan and 13 from India) were conducted from the three DIALOG+ implementing clinical sites (SCARF, KEH and JPMC). The interviews with pilot participants who had experience of DIALOG+ included 40 patients and 8 clinicians across both countries, out of which a majority were women and under 35 years of age (table 1). Basic sociodemographic characteristics of the naïve participant group are reported in table 2, and detailed service characteristics are highlighted in online supplemental tables 4–6. Out of those recruited, six participants withdrew from the interviews: two pilot patients and two caregivers from Pakistan and two pilot patients in India. Reasons included loss of contact (n=4, defined as more than three missed calls) with the research team as well as withdrawn interest in participating (n=2). To ensure a diverse perspective on their experience with DIALOG+, the pilot patients were replaced in order to secure a final sample of 20 participants from both India and Pakistan.

**Table 1:** Basic sociodemographic characteristics of patients and clinicians enrolled in the DIALOG+ pilot

Basic sociodemographic variables	Pilot participants (n, %)	
	Patients (n=40)	Clinicians (n=8)
Age (years)		
<35	20, 50%	5, 63%
36–49	13, 33%	2, 25%
>50	7, 17%	1, 12%
Gender		
Female	23, 58%	7, 88%
Male	17, 42%	1, 12%

**Table 2:** Basic sociodemographic characteristics of naïve participants (clinicians, caregivers and stakeholders)

Sociodemographic variables	Naïve participants (n, %)		
	Clinicians (n=16)	Caregivers (n=14)	Stakeholder group (n=13)
Age (years)			
<35	11, 69%	3, 21%	4, 31%
36–49	2, 12%	5, 36%	6, 46%
>50	3, 19%	6, 43%	3, 23%
Gender			
Female	12, 75%	6, 43%	6, 46%
Male	4, 25%	8, 57%	7, 54%

A majority of the recruited patients had been receiving treatment with a duration of less than 15 years (73%) under a mix of secondary and tertiary care facilities. Almost all patients recruited into the pilot were receiving psychiatric services at the tertiary level (98%). The majority of clinicians recruited into the pilot study were psychiatrists (50%) and psychologists (38%), with fewer allied mental health professionals, for example, social workers. Most clinicians had less than 9 years of experience (62%) working with patients who had mental health conditions, while only three clinicians were more senior practitioners, that is, more than 10 years of clinical experience (table 3).

**Table 3:** Treatment and service characteristics of patients and clinicians enrolled in the DIALOG+ pilot in Pakistan and India

Participants (n, %)			
Variables for treatment characteristics	Pilot patients (n=40)	Variables for service characteristics	Pilot clinicians (n=8)
*Services accessed for mental illnesses* *		*Professional background*	
Primary care	–	Psychiatric practitioners	4, 50%
Secondary care	15, 38%	Psychological practitioners	3, 38%
Tertiary care	39, 98%	Allied mental health professionals	1, 12%
*Treatment duration (years)*		*Work experience as clinician (years)*	
<15	29, 73%	<10	5, 62%
15–30	9, 22%	10–20	2, 25%
>30	2, 5%	>20	1, 13%

* Proportions exceed 100% in this category, as patients reported receiving treatments from multiple healthcare platforms.

Two main thematic clusters were included in the analysis, based on the aims of the study and the topic guide used. Within acceptability, four main codes emerged, that is, aesthetics, attitude, cultural consideration and relevance. Whereas, within feasibility, the open coding identified five main codes: training, application, intervention delivery, stakeholder involvement and time consideration (shown in figure 1). Key findings within these codes were organised within the categories of barriers, facilitators and suggestions with detailed participant quotes presented in online supplemental table 7.

### Perceived acceptability of DIALOG+

#### Aesthetics

Findings under this code included views on the accessibility of the DIALOG+ application based on its design (colours, font size and format) and language (local translations and phrasing of assessment questions)

*Barriers*: Both patients and clinicians from India and Pakistan commented on difficulties with the phrasing of domains and questions on the DIALOG+ application with potential to cause confusion in patients and extending consultation time for doctors with high work burden.

A few of them were finding it hard, difficult to adhrupthi [dissatisfaction], in Tamil mainly, in English it is fine but in Tamil the word adhrupthi [dissatisfaction], not everybody was able to understand it. (Pilot Clinician 2, India)

*Facilitators*: Patients from Pakistan found the design and colour of the application simple and suitable and found that the use of a touch screen helped them with recall. A clinician from India mentioned that the use of additional probes helped them to aid understanding of the domains within the DIALOG+ application.

*Suggestions*: The suggestion of adding visual elements and using simple conversational words in the local language font to aid comprehension of the scale was also mentioned by both pilot and naïve clinicians from Pakistan and India.

Rating scales, I had some difficulty in both English and Tamil. The patients- they were not able to- because they will be satisfied or dissatisfied each has separate categories, there is no range or any pictorial representation. (Pilot Clinician 3, India)

#### Clinician attitudes

The perceptions under this code referred to any attitudes perceived towards the DIALOG+ approach in terms of readiness or resistance to adopt DIALOG+ as part of routine mental health treatment or consultation.

*Barriers:* With regards to clinician readiness to adopt, one clinician from India felt unsure about conducting the first DIALOG+ session, while another from Pakistan believed sessions should be more doctor-centred, asserting that doctors understand patients’ conditions better. Integrating DIALOG+ into routine OPD hours was challenging, especially in high patient flow settings. Both Indian and Pakistani clinicians agreed that adhering to this protocol was difficult due to their caseloads.

See therapy or interview it is a process sometime in your mainstream work you cannot focus and especially in a psychiatric facility (Pilot Clinician 2, Pakistan)

*Facilitators*: Clinicians noted that while initial sessions took longer, subsequent sessions became more efficient, lasting around 30 min. Despite busy outpatient hours, clinicians from Pakistan found that DIALOG+’s structured design helped save time and organise sessions. Additionally, clinicians also highlighted its user-friendliness and proposed its usefulness for staff with less specialised training.

Individuals with minimum psychiatric training, especially our ward staff, I think they can use this. They already know how to use mobile phones and are aware of technology. (Pilot Clinician I3, Pakistan).

*Suggestions*: Clinicians proposed that an appointment system could prove valuable in carving out time for effective and focused discussions through DIALOG+ during busy outpatient hours. Naive clinicians from Pakistan also proposed the inclusion of categories (diagnosis, age, education level) to help decide which patient could benefit the most from DIALOG+ to minimise time wasted.

… it’s very difficult to use DIALOG+on everybody, so it will work- if you really want to help patients then give based on appointments and use DIALOG+. (Pilot clinician 2, India).

Both clinicians and caregivers also felt that sessions should be delivered more frequently (eg, on a weekly or fortnightly basis) and then tapered off as the frequency of visits for patients is reduced over time.

#### Clinical relevance

Perceptions under this code discussed the relevance of the DIALOG+’s content to people with psychosis and their clinicians while integrated in routine clinical practice.

*Barriers:* Concerns were raised about clinicians addressing non-medication issues, suggesting these be handled by specialised departments. Clinicians perceived that DIALOG+ might not suit patients in an active psychotic phase or with medication side effects. Caregivers worried that ‘personal safety’ questions could distress patients. Some clinicians also preferred traditional paper-based questionnaires over technological tools for clinical care such as tablets.

…patients with schizophrenia take medicines […] sometimes they feel lethargic because of that, sometimes they cannot focus, [they] are absent-mindedness, so their attention has to be captured, and that takes time. (Pilot Clinician 1, Pakistan)

*Facilitators*: Despite the barriers, most participants found the DIALOG+ domains relevant to the population. They emphasised the importance of focusing on areas such as mental and physical health, job situation, accommodation and personal safety, as these questions are not typically addressed in local services. In Pakistan, DIALOG+ was also recognised for its potential to develop problem-solving skills.

*Suggestions*: Clinicians from Pakistan and India felt DIALOG+ is suitable for educated or stable individuals with psychosis, ie, those on treatment and not experiencing active symptoms or medication side effects. One clinician noted that DIALOG+ could also benefit people with common mental disorders beyond psychosis.

it’s fine, according to me if educational criteria is set up in a proper way then I think it can be improved, because the patients coming to us, some are illiterate. (Naïve Clinician FGD, Pakistan)

#### Cultural considerations

Perceptions under this code referred to how the local culture (of Pakistan and India) affected the delivery or understanding of DIALOG+, particularly in light of existing levels of literacy and familial support systems.

*Barriers:* Caregivers from both countries demonstrated some dissatisfaction with not being involved in the DIALOG+ intervention along with their relatives. Reasons quoted included the lack of initiative and capacity by patients to independently make treatment decisions or follow through on consultation advice as well as a strong collective cultural norm that purports the involvement of family in the care of people with severe illnesses.

do you think our culture is in line with this methodology or approach, does our culture allow the patient to be independent? […] perhaps we would need to modify this slightly in line with our culture … caregivers in our culture are perhaps more involved and that the patients are perhaps not independent. (FGD Caregiver participant, Pakistan)

Clinicians also noted that excluding caregivers could lead to their disengagement, as they are often the first point of community support. However, participants from both countries mentioned that caregiver involvement might hinder patient progress if the patients faced abuse or felt unable to speak freely in their presence, or if sessions became dominated by family concerns.

While caregivers may have limited understanding, their proactive efforts often keep the patients’ experiences hidden. As a result, the patients who come to the facility are usually afraid… the DIALOG sessions should happen with the patients alone (FGD, Pilot patient, India).

*Facilitators*: Linked to the above, clinicians from India and Pakistan found caregiver involvement (even if brief, eg, 5 mins) during the DIALOG+ pilot as helpful towards maintaining adherence to the approach by patients as well as helping to address critical attitudes expressed by caregivers during busy outpatient settings.

Something like that, or that family member can do too, to motivate them, okay, good, they’ve done this, this week fully they’ve practiced this, or they’ve taken their medicine on time, this week fully they’ve set an alarm and gotten up on time, something like that (Pilot Clinician 2, India).

*Suggestions*: Participants had varying opinions on integrating family support during DIALOG+. Few caregivers and clinicians from India felt that only the patient should be involved or decide the caregiver’s involvement level. Conversely, some caregivers and clinicians from both countries believed family involvement could help patients adhere better to DIALOG+, make them more comfortable and provide realistic assessments, even if the engagement was brief (eg, 5–10 min with the primary clinician).

### Perceived feasibility of DIALOG+

#### Training

This theme included experiences by clinicians on the DIALOG+ training sessions provided. No barriers or facilitators were reported, but clinicians did provide targeted suggestions on better enabling them to deliver the approach in clinical practice.

*Suggestions*: While there were differing suggestions from professionals in each country, both identified the need to bolster the training with more practical guidelines. Clinicians from both countries recommended additional time for role plays, practising using the tablet, that is, selecting domains and using probes to generate and discuss action points for patients that were not as verbose.

When you are training the clinicians, they might want to spend some time looking at what their perspective of understanding is, and put those in as probes. (Pilot clinician 1, India)

#### Application and delivery of DIALOG+

This included experiences related to the process and delivery of DIALOG+ including factors around communication, physical constraints and assessments during the routine mental health consultations

*Barriers:* Despite having a tablet, Indian clinicians preferred using paper records for DIALOG+ due to usability challenges, whereas those from Pakistan noted that the lack of open-ended questions in the DIALOG scale affected communication with patients. Both patients and caregivers from India and Pakistan also commented on confidentiality issues within the crowded psychiatric services where DIALOG+ was being implemented as it probed on personal aspects of the patients’ lives.

*Facilitators*: A majority of clinicians from Pakistan and India noted that the structured discussion around domains helped them to acquire an improved relationship with and richer history of their patients’ conditions. Caregivers also shared this perception, adding that documenting the discussions could help clinicians in recalling individual clients and their concerns.

this is true… about memory… the tablet can perhaps help in recall and memory about the previous discussion with the doctors (FGD Caregiver participant, Pakistan)

*Suggestions*: There was general agreement that given the application’s benefits towards patient assessment, DIALOG+ could be adapted to also function as a diagnostic tool which includes monitoring and recording patient history, supplemented by caregivers.

I’m not a psychiatrist or a psychiatric social worker but it should be changed in such a way that it becomes a very effective diagnostic tool and it should be as diagnostic and as effective as possible. (Pilot Participant 6, India).

#### Considerations around time

This theme included experiences around how the time, duration and frequency of DIALOG+ aligned with and affected clinicians’ ability to conduct routine consultations within out-patient psychiatric settings.

*Barriers*: One of the main experienced and anticipated challenges related to the time taken for DIALOG+ sessions in the context of busy and space-limited outpatient settings. This finding was reported by multiple clinicians in both countries, quoting high patient loads and limited individual time with patients as a predominant barrier to the adoption of DIALOG+ in routine clinical practice (as it required more targeted one-on-one support with each patient).

Having more than 20 patients in an outpatient department is not going to be easy. It will be a crowded clinic. We don’t have a system where people come at specific times for consultation … here it doesn’t happen (Naive Clinician, India).

*Facilitators*: However, clinicians from India proposed that the experience of conducting subsequent sessions can aid in better time management of implementing DIALOG+ in routine outpatient settings (eg, from 60 to 45 mins to 25–30 mins). Patients from both India and Pakistan felt that the DIALOG+ sessions were delivered in an appropriate duration which saved time and brought more structure to their sessions.

[The] tablet is good. Because it can quickly determine what you need. (Pilot Participant 5, Pakistan).

*Suggestions*: Most clinicians emphasised the need to keep an appointment system in place for patients coming in for DIALOG+ sessions to better prepare the clinician with additional workload as well as support the integration of DIALOG+ more seamlessly into the organisational setup. There was interest from both a clinician and caregiver from India to prefer a higher frequency of DIALOG+ sessions (eg, fortnightly) but noted this should depend on the patient’s concerns and the clinician’s availability.

#### Stakeholder involvement

This code summarises views by clinicians, patients and caregivers on the participation of patients and family members in the DIALOG+ approach.

*Barriers*: Many clinicians from India and Pakistan found motivating their patients to independently make treatment decisions or actions to improve their condition care challenging. Many referenced a cultural belief equating ‘medical professionals as equivalent to God’ (Pilot Clinician 2, India) indicating the burden of recovery predominantly placed on clinicians and medical practitioners. Similarly, patients reported not being able to follow-up on action points agreed on.

they gave me homework but I didn’t follow through. I’d keep it in the cupboard on the first day to work on it daily but I’ll end up watching television. I should have done it. (Pilot Participant 8, India).

*Facilitators*: Some clinicians from India appreciated the approach as it facilitated independent decision-making on the part of the patient and improved engagement between the clinician and their patients.

They should be an individual person independent we cannot keep pampering and making decisions for them. So they should learn to do this on their own. (Pilot Clinician 3, India)

*Suggestions*: There were varying suggestions from clinicians on how to improve patient involvement, including taking the time to build their patients’ confidence in undertaking actions to more directive advice such as placement within rehabilitation hostels or asking their caregivers to help them.

## Discussion

We conducted a qualitative study with clinicians and caregivers of people with mental illness as well as participants from the DIALOG+ pilot study, with the aim to assess the feasibility of implementing this approach in routine clinical care and the acceptability of the intervention with clinicians and patients with psychosis from India and Pakistan. In general, while there was consensus on the benefits brought on by DIALOG+ to session structures, the capacity of clinicians to implement the approach in their routine practice was highlighted as a significant barrier to its adoption in clinical practice. Many suggested more comprehensive and practical training programmes to help clinicians with effective and efficient delivery as well as appointment systems for allocating dedicated time for meaningful discussions with patients. However, this calls to question the feasibility of implementing the approach in routine outpatient hours. Solutions to mitigate clinician work burden require longer-term actions such as institutional ownership and system strengthening initiatives such as task-sharing with non-specialist providers and recruiting more mental health professionals to address the resource deficit.^27^ Ongoing engagement of key stakeholders such as service directors and clinicians at all stages of research design can also facilitate their investment in contextual solutions to mitigate the challenges associated with clinical workload.^28 29^ While this pilot was a step in the right direction, further investigation is required in identifying successful approaches to building ownership in healthcare policy makers to adopt patient-centred psychosocial care within routine psychiatric care and creating systems for reducing provider strain and burn-out.

When it comes to the acceptability of psychosocial approaches, global and regional literature underscores the importance of cultural adaptation when using digital interventions in healthcare interventions.^30^ Multiple clinicians and caregivers heavily purported the importance of doctor-centred assessments and family involvement to improve the reliability of the assessment process and patient adherence. While family involvement in psychosocial care has been associated with improved social capital and collaborative care, the assumptions behind these perceptions highlighted limiting cultural beliefs around patients’ decision-making abilities for the treatment of their psychosis.^31^ It’s important to note that the reduction in clinician-centredness, which clinicians cited as a barrier, is actually an intentional aim. This is because DIALOG+ is designed to make psychiatric care more person-centred by increasing patient participation in decision-making and treatment planning over sole clinician intervention in consultations. It is clear that more work needs to be done to promote the benefit of patient-centred services and the perception that service users can be empowered to make these decisions more autonomously. In order to strike a more equitable balance between patient autonomy, caregiver influence and clinical expertise, the importance of patient-centred care should be promoted as part of the ongoing scale-up of DIALOG+ in the South Asian context. This inquiry also aligns with key research priorities produced by the Lancet Psychiatry Commission on Psychoses in the Global Context and contributes to locally led evidence on psychosocial care for people with psychosis in LMICs.^32^

## Conclusion

This study shed light on the nuanced interplay between feasibility and acceptability in the implementation of the DIALOG+ approach in urban Indian and Pakistani psychiatric settings. While perceptions on the effectiveness of DIALOG+ in improving patient outcomes were not a key aim in this research paper, other implementation factors of the approach, for example, structural benefits and addressing challenges related to clinician capacity and cultural perceptions were outlined as critical for enhancing the adoption of patient-centred psychosocial approaches within routine psychiatric practice. Further research and ongoing collaboration between mental health services and researchers are crucial to identifying and evaluating effective strategies for enhancing institutional ownership, supporting clinicians with manageable patient workloads and enhancing shared decision-making power with the goal of strengthening mental health services and outcomes in South Asia.

## Supplementary material

10.1136/bmjopen-2024-091852 online supplemental file 1

## Data Availability

Data are available upon reasonable request.
