# Supplementary material for: Exploring the feasibility and acceptability of DIALOG+ (a structured digital communication tool) in strengthening psychiatric care in India and Pakistan: a qualitative pilot study
Source: BMJ Open. 2025 Aug 12;15(8):e091852. doi: 10.1136/bmjopen-2024-091852 (PMC12352267; doi:10.1136/bmjopen-2024-091852)
Supplement: online supplemental file 1 [file bmjopen-15-8-s001.docx]

| **Socio-demographic variables** | **Participants (n, %)** |
| --- | --- |
|  | Naive Clinicians (n=16) |
| Age (years) | |
| <35 | 11, 69% |
| 36-49 | 2, 12% |
| >50 | 3, 19% |
| Gender | |
| Female | 12, 75% |
| Male | 4, 25% |
| Professional Background | |
| Psychiatric practitioner | 6, 37% |
| Psychological practitioner | 7, 44% |
| Allied mental health professionals | 3, 19% |
| Work Experience (Years) | |
| <10 | 12, 75% |
| 10 - 20 | 3, 19% |
| >20 | 1, 6% |

*Table 4. Detailed participant characteristics of naïve clinicians (not exposed to DIALOG+) enrolled in the study*

| **Socio-demographic variables** | **Participants (n, %)** |
| --- | --- |
|  | Caregivers (n=14) |
| Age (years) | |
| <35 | 3, 21% |
| 36-49 | 5, 36% |
| >50 | 6, 43% |
| Gender | |
| Female | 6, 43% |
| Male | 8, 57% |
| Relationship to Patient | |
| Parent | 5, 36% |
| Sibling | 5, 36% |
| Spouse | 2, 14% |
| Child | 1, 7% |
| In-Law | 1, 7% |
| Experience of Caregiving (Years) | |
| <10 | 6, 43% |
| 10 - 20 | 8, 57% |

*Table 5. Detailed participant characteristics of caregivers enrolled in the study*

| **Socio-demographic variables** | **Participants (n, %)** |
| --- | --- |
|  | Stakeholder Group (n=13) |
| Age (years) | |
| <35 | 4, 31% |
| 36-49 | 6, 46% |
| >50 | 3, 23% |
| Gender | |
| Female | 6, 46% |
| Male | 7, 54% |
| Professional Background | |
| Psychiatric practitioner | 4, 31% |
| Psychological practitioner | 2, 15% |
| Business Management | 2, 15% |
| Other | 5, 39% |
| Work Experience (Years) | |
| <10 | 5, 38% |
| 10 - 20 | 4, 31% |
| >20 | 4, 31% |

*Table 6. Detailed participant characteristics of the mixed stakeholder group enrolled in the study*

| **Main**  **Codes** | **Barriers** | **Facilitator** | **Suggestion** |
| --- | --- | --- | --- |
| Aesthetic | Clinician: "Rating scales, I had some difficulty in both English and Tamil. The patients- they were not able to- because they will be satisfied or dissatisfied each has separate categories, there is no range or any pictorial representation. So when I attend to patients who have had only primary education or no education at all, we are not able to point out a particular number. So I had that issue. So if this is corrected maybe we can have an even more clearer idea." (India)  Caregiver: "There were a few questions in this, I am unable to read these due to poor eyesight" (Pakistan) Patient: "It would have better if there were pictures" (Pakistan) | Clinician: "This seems to be more suitable for doctors" (India) Patient: ".. you can make it more attractive. Some people will like it more if it is attractive [...] Like… put a heart like thing and two hands like this.." (India)  Clinician: "the font size is also very convenient that if someone's eyesight is weak or even if its not they can look very easily…secondly if you keep it at such a distance the patient can look at it very easily" (Pakistan) | Clinician: "Pictorial representation of anchor points will help patients" (India) Stakeholder group: "When you add a some picture of it no ma`am, it will be easy to understand." (India)  Stakeholder group: "It would be nice if music played in the background, to calm our minds" (India)  Patient: "please change the colour pallete to Pink, Grey, Maroon and Red, this would enable better readability for everyone, This is already known by everyone." (Pakistan) Clinician: "For graphical representation…if this is a scale, for instance, show them in this way, then it may be difficult to make them understand, that now where is the issue, or let us know via pictures that now we are going to talk on this domain, or hobbies, so on the side you can just put pictures related to hobbies, like readings books, or this, in this way so that patient would get more involved in this" (Pakistan) Patient: ".. introduce language that allows everyone to understand and make it easy [...] And you can bring a new type of visual image or format." (Pakistan) Clinician: " think it’ll be nice if they have an audio also, because illiterate person do not know how to read, so if there's an audio wherein the question is being read out, it’ll be more helpful" (Pakistan) Caregiver: "I think it should include reviews by caregivers, plus have a space for adding patients habits e.g. what they do at home" (Pakistan) |
| Attitude | Patient: "Like I could not follow up, they gave me homework but I didn't follow through. I’d keep it in the cupboard on the first day to work on it daily but I'll end up watching television. I should have done it" (India)  Patient: "No there is no solution to this. For instance, I’m saying over and over I don’t have a friend or any activities, and nothing has been resolved in this area, they just keep asking questions." (Pakistan) Clinician: "Here, the concept of community care does not exist in Psychiatry. And this concept of community care deals with rehabilitation measures for patients, which helps them in the long run. Unfortunately, we are unable to do that here. I, as a doctor, can prescribe medicines and arrange psychotherapy for patients at most. But other than that I can’t help patients in things like finding friends, solving accommodation issues or financial issues" (Pakistan) | Clinician: "I think this could be- we can able to precisely collect what their needs and then so that we can go further on that to improve their- whatever the things they need. I feel it's very precise I would say." (India) Patient: "Sir, I have been taking medication for twenty years, if this intervention had been there when I was younger, I would've benefitted from it greatly, and it would've helped improve my concentration as well." (India) Patient: "It has broadened my mind as to how I can talk to the Doctor and what are the aspects I can talk to the Doctor Instead of only talking about my Physical Health and Mental Health. So many other things are there… these things also I can talk to the Doctor I was not aware of that" (India) | Clinician: "You need to attract the attention of a lot more clinicians in a more positive light. And if we are able to achieve that over the next 6 months or so - I think getting it into – I think there will be an evolution of people starting to implement it outside the trial period" (India) Patient: "DIALOG+ is definitely required for all patients. It will useful for anyone coming for the first time, instead of just prescribing medications, giving them this space to focus on their issues will help understand their issues and find solutions." (India)  Clinician: "Yes ofcourse. If DIALOG Plus can somehow be connected to a system of a tertiary setup, like in this hospital everything is being operated through buttons. For instance, if I am thinking of Psychotherapy or nutritional support for a patient, perhaps there could be a system where I enter a code, it develops a referral, and an sms to the patient automatically." (Pakistan) |
| Clinical Relevance | Clinician: "Rating of either very satisfied or slightly satisfied is difficult. All what they know is I’m good or I’m not good’ (India) Clinician: "So when I attend to patients who have had only primary education or no education at all, we are not able to point out a particular number. So I had that issue" (India) Patient: "Problem is many people- poor people they cannot afford [...] Coming here they will afford, everybody should have a smart phone- smart phone now many people are having, I don't think- many people are having- 80% - I dont know but some people will not be having[smart phone]" (India)  Patient: "in a few places you have asked about job prospects etc… Jobs etc… this may not be applicable for most patients and hence this section would often remain empty… neither positive nor negative… therefore perhaps the structure should perhaps be revised" (Pakistan) Caregiver: "the respondent or patient will get irritated.. the way the other person shared, patients are at times very scared.. they may become afraid and go into a sort of shell….most patients are under immense stress and at times dont have the general capacity to understand and express clearly" (Pakistan) Clinician: "Most of them have flight of ideas, most of their speech is irrelevant, they are struggling with grasping things, facing problem so one reason is to secure time, cognitively they don’t have grip to a level to understand this app properly" (Pakistan) | Clinician: "The patients engaged in the DIALOG+ sessions with more enthusiasm given that they felt heard and considered. This, they said, improved the outcomes from the process" Patient: " 4 step approach is good, the basics is the patients are able to make their own choices, will not force anything upon them because everything is their choice they choose it. So that is good."  Clinician: "2 of my patients were a little- their education level were also a little- college-educated and their symptoms are also not very interfering so they were able to catch it up and then next session onwards it was going fine"  Clinician: "Exactly, the record will also get maintained, and we will have starting history and information in collective form, further in implementation if we have to add or subtract so on it’s basis we can select very easily" Clinician: "Because if we take physical sessions we don’t have any scale for improvement check, or no scale to check progression changes, so this scale is very good" Clinician: "That is very good, very comprehensive. If something is slipping from your mind, it does not let you skip it. It indicates to you that this is remaining, this is not done yet.. and this helps narrow down a lot."  Patient: "DIALOG plus is better, it looks very generally, rather than involving – you don’t get stuck because you can’t understand concepts of psychology" (Pakistan) | Clinician: "This thing is mostly- uh some educated patients will be fine with these things" (India) Patient: "Some people may be slow, they may not know English, and some people may not be educated [...] Suppose they’re not educated, we have to cater to that, take some time." (India) Clinician: "I don’t feel it is relevant to psychosis patient it will be common to every patient every patient can talk about these things when they come, actually it is useful for them to get some insight of their own problems it will be helpful" (India)  Caregiver: "in a few places you have asked about job prospects etc… Jobs etc… this may not be applicable for most patients and hence this section would often remain empty… neither positive nor negative… therefore perhaps the structure should perhaps be revised" (Pakistan) Clinician: "Would be easier if it get categorise to apply upon which patient… then wouldn’t be applying on each patient... because the patient with psychosis their cognition won't be intact so we will not be applying on them, so it would be easier and also time saving when it get categorized" (Pakistan) Clinician: "I felt that this one domain related to education was missing… Because this point for education is very important.. Because this is a moderator between a child and parents" (Pakistan) |
| Cultural Consideration | Clinician: "If caregivers are involved they will take over the session and will start talking about their difficulties with the patients" (India) Clinician: " I find a little-a- bit of a divide between the DIALOG+ and our cultural value it is very [...] person centric or patient centric and our culture is a collectivist society, this is developed in UK which is more an individualistic society." (India) Clinician: "patients find it difficult to think of action points on their own and they had to prompt every time and the clinician mentions that it is a cultural thing to just listen to what the clinicians say and obey" (India)  Caregiver: "the question as to whether has there been any situation where someone may have ever teased her…. i was about to ask the doctor to skip this question..since we never let my sister venture out alone unsupervised.. she is always chaperoned by a family member, she looked at me blankly as to what should be the answer to this? then the doctor filled in the answer on himself" (Pakistan)  Caregiver: " do you think our culture is in line with this methodology or approach, does our culture allow the patient to be independent? someone earlier spoke about how they accompany their sisters, their daughters so do we have such an environment in our clinics?" (Pakistan) | Clinician: "Family members supported patients to complete the action points" (India) Clinician: "They take 5 mins extra time to explain a gist of what happens to the caregivers and it is helpful" (India) Stakeholder group: "This is the structure of families in india. We have more of this compared to the west that is why we have been able to show better outcomes for intervention with schizophrenia." (India)  Clinician: "It’s positive for the patient, because it enhances their understanding. But for patients with schizophrenia, we have to keep their social and cultural setting in mind and ensure that those aspects are strong. Because their sole support is their family. Hence here, I think equal participation and engagement should be more effective" (Pakistan) Patients: "The freedom here is that we have parents, friends, someone or the other and a bigger sense of community" (Pakistan) | Clinician: "When the caregiver tends to intervene, they overshadow the patient and talk about their own needs and expectations. When the patient creates their own action plan, the caregivers should be supportive and respectful of their efforts" Clinician: "But it might be useful if we either find a very cultural way of defining the anchor points in a specific way of defining these points." (India)  Clinician: "There is confusion between friendship and partner that we have to explain.. So either you keep spouse separate which can be skipped, we know who is unmarried, so keep the relationship with the spouse separate from the family , and keep the friends separate" Caregiver: "..a better way that would perhaps get better insights, real insights, and in turn enable you to help patients better would be to perhaps include caregivers given our cultural differences" (Pakistan) |
| Training | Clinician: "one needs to do a lot of role playing before going into the field, so that you have it memorized in your mind you wont have to explain what 5 means what 6 means" (Pakistan)  Clinician: "See the kind of training that I got - well I was invested in the project. So I didnt really any problems in adapting. But the Naive clinician may not want to do or have anything to do with it. So you are gonna actually try and tell - sell the topic to them." (India) | Clinician: "At least 3 observation will be fine- 3 observation will be fine and 1 mock is fine, and after observation the mock will be fine." (India) Clinician: "See the kind of training that I got - well I was invested in the project. So I didnt really any problems in adapting." (India)  Clinician: "*Patient1* said what is this show me as well. So I put the computer in front of patient, I had to bend to see the questions, or if I had a printout I could ask questions so that is easy just a pilot, to reinforce role playing a bit first and everyone should learn each and every question so that they don't need to look at the screen" (Pakistan) | Clinician: "But one thing, what I would recommend during the training for the clinician is. We must focus a lot on the four step process [...] We must spend at least 20% or like 25% of the time going through that process" (India) Clinician: "when you are training the clinicians, they might want to spend some time looking at what their perspective of understanding is, and put those in as probes." (India) Clinician: "So using the tab before - so just give them you know - give it to them for one week or so and say okay try and use it everyday. And they might use it just on one or two people or maybe little more but att least gets them the idea that you know it can be - it’ll encourage them to think more positively about it is what I’m suggesting" (India)  Clinician: "When you train newcomers you make them do a lot of role-play.. And keep a limit of how many sessions to do before they can go on field" (Pakistan) Clinician: "Individuals with minimum psychiatric training, especially our ward staff, I think they can use this. They already know how to use mobile phones and are aware of technology, and this is tablet based. So they should be able to use this with ease." (Pakistan) |
| Application | Clinician: "I will make sure- make the eye contact with the patient when they are talking but sometimes- while I am using this thing [tablet] sometimes I miss the eye contact that is the thing." (India) Clinician: "The other challenge that I faced was to try and type in into the tab right? So that was another challenge that I faced and I think this was perhaps only because i was not used to it." (India) Caregiver: "You are trying to make it easy for them by providing the app on the mobile, but instead it is making them [patients] idle" (India)  Clinician: "...I met patient and asked close-ended questions, with yes or no, then focus gets limited. like if I asked his difficulties that what happened… the communication goes in detail.. and facilitates conversation, but in this way communication would get short and can not take out more things" (Pakistan) Clinician: "If I want any patient to hold the tablet, read the statements on their own in front of me and then do the scoring, I’ll give them the tablet. Often times patients are not willing to touch the tablet, or frequently miss touch the tablet." (Pakistan) | Stakeholder group: "I think the patient will feel quite empowered through this app because we feel that finally there is somebody paying this much attention to me to know very specific things rather than just medical" (India) Patient: "some people may have issues regarding their family situations, or with respect to their medications, these issues are covered by the questions provided in this app, and it becomes much easier to address these issues openly as well." (India)  Caregiver: "Since it serves as a documentation tool as it allows us to record our discussions with the doctors, and in case we are unable to remember or recall anything, this app will have the complete details… the patients will be able to recall their discussions with the doctors" (Pakistan) Patient: "This thing [tablet] is very simple and anyone can do it easily. Science has made great breakthroughs. If you get familiar with this [tablet], everyone is familiar with this, [...] this thing can bring new changes and it should be used, it’s a good way to keep the patient engaged in a positive way and communicate better." (Pakistan) Clinician: "...very good to organise, it's like a journal which we maintain that which step to keep follow so what to do next" (Pakistan) | Caregiver: "Some people are there who has fear in technology. We should make that people understand, and they can use it." (India) Clinician: "the app should perhaps have somehting extra, perhaps additional pages and features, general knowledge, reading etc. [...] asking questions about medical conditions is important i understand but there is only a 50 50 chance that the patient may improve by talking about the conditions, however talking about daily routine is perhaps more likely to take the patient towards improvement" (India)  Clinician: "Questions can be kept separate for patients aged 3 - 15, perhaps in the form of an age selection at the top or start of the app…there can be separate questions according to gender, age, married or otherwise" (Pakistan)  Clinician: "the fewer the components, the more user-friendly it will be since we have limited time." (Pakistan) Clinician: "Structuring the app’s language in a way that its question, the presentation of options. The presentation of scoring in an easier manner." (Pakistan) Caregiver: " the perspective of the caregiver should also be gauged or included… and perhaps these must also be visible to the doctor at the time of assessment, example a patient says im completely fine, the caregivers ratings and views may perhaps be different." (Pakistan) |
| Intervention Delivery | Clinician: "It’s not only with DIALOG+ it always happens with patients, you say something, you give them homework, they don’t remember, you make them write somewhere" (India) Patient: "It’ll get crowded, else we can have it. You have to see so many patients after me right thats why its not possible in OPD" (India)  Clinician: " If one changes the number of patients here and keeps 15 doctors, that would be challenging [...] Unlike a Pilot project, this experience will be entirely different. Where will the patients come from, how long can the doctors be on these permeable terms, how long will the FCD be, ofcourse all of this will be challenging. Having 15 individuals working uniformly on a standard pattern will be very challenging" (Pakistan) Clinician: "not every patient can get psychotherapy. If a patient has issues with their job and they are unable to find a new one, we know that their complaints are going to be similar in the next visit [...] If a patient has issues with their accomodation and they can afford a new one because of financial constraints, we know that these problems will be there in the next visit too. Hence here is a limitation for us since we can’t do much." (Pakistan) | Clinician: "Now this particular program demonstrates to me personally that you can bring in a structure to the conversation which is happening with the client. I would be asking the very same questions during a clinical review within a routine OPD. But I might not be covering all the domains that are cited out there" (India) Clinician: "The intervention helped the patients come up with their own solutions and expectations from the sessions as opposed to waiting for the clinician to guide them, which was useful" (India) Patient:" I’m usually scared of doctors,... But that fear went away with each session, I felt free.." (India)  Clinician: "I get their feedback on how their previous week was. This helps in understanding whether the patient remembers the last session; whether they practiced the homework assigned to them; if they did where did they find it difficult or what were they able to do easily [...]This helps in understanding whether the client remembers the discussion from the last session" (Pakistan) Clinician: "Sometimes it happens that a patient comes and since morning you are so exhausted and you even have to take a patient- for which your mind isn't even working so for that this pre-made well designed app in front of your eyes there is no other app to do, one opens the tab and it comes in front-so in that way i find it very convenient, there are no open ended questions in this either, so you don't have to think yourself, and usually when you read the app" (Pakistan) | Clinician: "Should be an add-on to the clinical services, it should be provided upfront so it shouldn’t be a thing that's being done by clinician as such but it could be a one-step ahead or in front of the- before seeing the patient itself" (India) Patient: "Thats what I want to say something-one thing you have tele-psychiatry or through mobile, you can administer the app from here- you can- the doctor can administer from his room to a patient when he has time" (India)  Caregiver: "It should have been done in a particular segregated room… with a proper detailed session and discussion, in a secluded safe environment, with no other persons present in the room [...] this was the case for my brother and I know full well nervous he becomes in such situations. . where the questions were being asked to him in the presence of other people" (Pakistan) Clinician: "We definitely write things on paper, because patients take that paper home so that they can remember. Be it changes in medications, or strategy or psychotherapy, we have to document that separately" (Pakistan) |
| Stakeholder, involvement | Patient: "It's not that they don't know anything, their parents or caregivers tend to interrupt, and so they aren't able to express their opinions freely. A lot of them feel scared." (India) Caregiver: "..have to always remind them. They will do the homework. Then only they will do." (India) Patient: "Some people may find it difficult to follow through with the plan they agree to follow, however, I feel that if it were explained to people their caretakers, then they would help the patient follow it." (India)  Caregiver: ".. the caregivers are not directly involved in this study?? one thing that i keep thinking about this study that you are conducting and presenting, is as to whether patients are independent or capable enough in terms of capacity to be able complete these steps" (Pakistan) Clinician: "We encourage the patients in every way. But some patients are not willing to do it by themselves." (Pakistan) | Clinician: "..here it is a direct 40 minutes interacting only with the patient. So we have firsthand information from the patient, what he wants, and we focus on that. So that’s good here." (India) Clinician: "Yes, that's the difference here because the action plans were made by the patient not by the caregivers. So the only job as a caregiver is to respect and support the action plans made by the patient." (India)  Clinician: "Here, the patient reads a question, tries to think about it and understand it, answers it, then answers the subsequent questions that are being asked. So this is more engaging" (Pakistan) Clinician: "But for patients with schizophrenia, we have to keep their social and cultural setting in mind and ensure that those aspects are strong. Because their sole support is their family." (Pakistan) | Clinician: "But in this, we are also involved in that it makes the patient feel more needed and more wanted. So, at the end of this pilot I would visibly see changes in many people." (India) Clinician: "No, definitely caregivers should not be [...] present in this room in the interview is going on because they are very protective when the caregiver is there, so they are not ready to complain, not ready to open their-uh personal problems" (India) Clinician: "Having an appointment system not only prepares the clinician but also the patient to get ready to discuss his/her concerns" (India)  Caregiver: "At least bi monthly feedback from family and caregivers must also be collected to guage the ground situation and reality of improvement for the patient" (Pakistan) Clinician: "Should be there is one session with both the patient and their family [...] because family is the support system; the stronger the support system, the sooner the patient will recover" (Pakistan) Caregiver: "With a complete database and history, allowing doctors to access past notes and history at the time of dicussions, the caregivers also can have access to enable them to track progress" (Pakistan) |
| Time Consideration | Clinician: "I think the caseload- with the given caseload in our OPD, [...] it’s very difficult to use DIALOG+ on everybody," (India)  Patient: "What I did not like was it was too infrequent once in a month is not enough" (India)  Clinician: "Our OPD is usually very busy and is also very hectic. Therefore, there is not much time left to discuss every domain, such as what are the issues at the workplace, or what kind of relationship issues one is facing. Mostly, our discussion revolves around medications; but, we are not able to discuss this in detail" (Pakistan) Clinician: "So I think this structure is time consuming, and patients visiting Hospital 1 expect that we hear their problems and respond immediately" (Pakistan) | Clinician: "The first initial session will take around 40 minutes however the subsequent sessions will come down to half an hour" (India) Clinician: "It’s not about asking about their symptoms alone. We’re talking about specific areas in their life, specific things, so we’re spending adequate time" (India) Patient: "If we meet once a week, it would be easier for them to give us ideas on how to solve our problems more effectively." (India)  Clinician: "in start of implementation, it will consume time, would be difficult in terms of time, and conveying to patient, but will get use to of it with time, then gradually these hurdle of time consuming will decrease" (Pakistan) Clinician: "Our time gets saved. I personally feel that questions in DIALOG Plus have been formulated keeping in mind the psychosocial model" (Pakistan) | Clinician: "First 3 visits can be done once in 2 weeks and later, if the patient is still seeking help, once a month could work" (India) Patient: "I feel that it is a beginning and it can be enhanced or streamlined more to be more effective its what I feel" (India) Caregiver: "perhaps the sessions can be planned in such a manner after discussion with family and caregivers.. and they coordinate to ensure that the condition of the patient is perhaps suitable to answer" (India)  Clinician: "Between 1 to 3 months, to atleast give time to the intervention we’ve done with the patient. If I ask a patient who visited last week if they’ve found a job, I know that they wouldn't have one even in the next week’s visit." (Pakistan) Clinician: "If the number of patients are less, and human resources are structured in a way that can schedule consultations for the patients. I don't have many people. If I did, then this would have been a very good set-up." (Pakistan) |
| *Table 7. Detailed participant* *quotes of key barriers, facilitators and suggestions by codes* | | | |

**Supplementary File 1 – Interview/Focus Group Discussion Topic Guide and Questions**

*NB: The focus group will follow a 30 minute Workshop where participants will be shown the DIALOG+ app (the App / mock-ups)*

*[Introduction]* Thank you all for coming today and for taking part in this focus group where you have been introduced to the DIALOG+ app. This is a research project looking to make changes and improve this app so that it is suitable for use with people with psychosis. Your contributions here will be very useful for further developments. [Explain how app works – rate each life domain, 4 step approach, actions]

I’m [researcher name] and I’ll be discussing your opinions of the current DIALOG+ App, and ways we could improve it. This is my colleague [researcher name] s/he will be taking notes to help me and asking a few questions too.

The purpose of this focus group today is for us to get some honest feedback about DIALOG+. We’d like to hear your views on what you like and dislike, and whether you think anything should be changed or added. We would like to develop the intervention to make it suitable for people with psychosis so it’d be great to hear your suggestions, improvements etc. We would of course like to hear your views on anything else you think is important about the intervention as well.

To save us writing down everything we are audio recording this group but this will be transcribed and identifying features will be removed and the tape destroyed afterwards so everything you tell us will remain confidential and anonymous.

Can I also ask that you do not repeat things you have heard here. We want people to feel comfortable being open and honest so we ask that you respect that confidentiality.

Are there any questions before we get started?

*[Ground rules]*

- Purpose of the focus group-group discussion on DIALOG + that will be audio-recorded and researcher taking notes.
- Remind no right/wrong answers but views and opinions, we are interested in finding out about opinions of the DIALOG+ intervention
- Reiterate what data is used for, confidentiality and ask others to treat all discussions confidentially.
- Request that individuals do not use names of others or of specific services, if possible when talking about their experiences. But if you do then these will be removed when the discussion is transcribed.
- Remind participants that they can use pseudonyms or nicknames if they do not want their names appearing on the recording
- Ask people not to speak over each other but one at a time.
- Remind of time (60 mins max)
- Reminder that individuals can leave at or have a break at any moment

*[Warm up question]* “We’ll start off by asking everyone to introduce themselves.”

1. *[general impression]* “OVERALL WHAT DO YOU THINK OF THE DIALOG+ INTERVENTION?”

- What did you like/dislike?
- Do you think it will be helpful or not helpful for people with psychosis?

1. *[rating scale]* “WHAT DID YOU THINK OF THE RATING SCALE FOR THE 11 DIFFERENT AREAS (MENTAL HEALTH, ACOMMODATION, JOB SITUATION etc.)”

- What did you like/dislike?
- Was the wording of the question “how satisfied are you….” Easy or difficult to understand, why?
- Do you have any suggestions for how it could be improved?
- Does this accurately cover everything you consider to be important to you in your life / the lives of people with psychosis?
- Are there any other areas that are important to you which should be added rating scale?
- Do you have any suggestions for areas to be removed?
- Was it easy or difficult to think about the areas covered and apply satisfaction ratings?
- How often would you like to do the rating?

1. *[CHOOSING DOMAINS] “WHAT DO YOU THINK ABOUT SELECTING UP TO THREE AREAS TO DISCUSS IN FURTHER DETAIL DURING THE MEETING WITH YOUR CLINICIAN?”*

- Do you think it would be easy or difficult to discuss the areas with a mental health worker?
- How many areas would you like to discuss?
- What areas would you like to discuss with your clinician / mental health worker in more detail?
- Is there any area that you would find it particularly difficult to discuss with your clinician or mental health worker?
- Is there anything that you would like to discuss that was not an option?

1. *[4-step approach]* “ASK FOR OPINIONS AND IMPROVEMENTS” (go through each step one-by-one

- What did you like and dislike about the 4-steps
- Is there anything missing from the conversation?
- Would you find it easy to think about small improvements that could be made to help your situation?
- Do you understand what is meant by resources (e.g. your resources, clinician’s resources)?
- Do you think it would be easy to identify your resources, the clinician’s resources and the resources of others?
- What did you think about recording actions?
- Can you think of ways this might be improved?

1. *[barriers and facilitators]*  IS THERE ANYTHING THAT WOULD MAKE IT EASIER OR MORE DIFFICULT TO USE DIALOG+ WITH YOUR CLINICIAN OR YOUTH WORKER DURING YOUR APPOINTMENT?

- What would make it easier?
- What would make it difficult/what are the barriers?
- Do you think there would be sufficient time in your appointments?

1. *[look of the current app]* “IN GENERAL WHAT DO YOU THINK OF THE LOOK OF THE APP?”

- What did you think about the colours used in the app?
- Did you like the font (writing)?
- Was the size of the font big enough?
- Does the wording of the questions make sense to you (e.g. “satisfied”)
- Is there anything missing from the design that could improve it or makes things clearer?
- Is there anything you would like to change?

1. *[how meetings changed]* WHAT DO YOU THINK ABOUT USING A TABLET IN YOUR APPOINTMENT?

- What changes do you think it would make to your appointment?/ Relationship with clinician?
- Do you think the conversation or areas covered in your appointments would be different? How? Why?
- If yes, how do you feel about the changes?

If no, are there things that you would like to have changed?

- Do you think you would talk to your clinicians differently? In what way?
- Would you want reminders between sessions about action points, e.g. text messages, emails etc.

1. *[Summations]* “IF YOU HAD TO SUGGEST JUST ONE KEY IMPROVEMENT FOR DIALOG, WHAT WOULD THAT BE?” [Warn the group that the focus group is nearly over and ask for final suggestions of comments. Go round the group and ask everyone to say one key improvement or one final thought. Following this thank the participants for their participation in the focus group.]

**Supplementary File 2– Informed consent form**

**INFORMED WRITTEN CONSENT SCRIPT - WP1: DIALOG+ PILOT TRIAL - PATIENTS**

**Study Title:** Improving outcomes for people with psychosis in Pakistan and India – enhancing the Effectiveness of Community-based care (PIECEs)

**What you should know about the study:**

- You are being asked to join a research study
- This consent form explains the research study and your part in the study
- You are a volunteer. You have the right to choose whether to participate or not participate in this study. If you decide to participate and later change your mind, you may do so without any penalty or consequences.

**Purpose of research project:**

My name is ________. I work with IRD/SCARF. This research project is interested in exploring a new method to help patients with psychosis. This method is called DIALOG+ and involves using a tablet computer to guide your regular treatment sessions between you and your mental health professional.

In the DIALOG+ intervention, patients are asked to rate their satisfaction with eight life domains (mental health, physical health, job situation, accommodation, leisure activities, friendships, relationship with family/partner, personal safety) and three treatment aspects (medication, practical help, meetings with professionals). The tablet allows patients to be more actively involved in the meeting, with the tablet easily passed between the clinician and patient. The ratings are followed by a conversation to identify the patient’s existing resources that can be used to address the concerns raised and work to improve a patients quality of life.

In this study, we wish to pilot and try out the existing DIALOG+ intervention over a three month period.

**What you will be asked to do in this study:**

We are inviting patients who regularly see their mental health professionals at an outpatient clinic to try this new treatment/intervention. If you are willing to take part, you will first need to agree and sign 2 copies of this consent form. One will be given to the study coordinator and one to you.

In this pilot study you are being invited to use the DIALOG+ treatment with your mental health professional for 3 months during each of your visits to see your professional. Each visit will last around 45 minutes. At the end of 3 months, we would like to know your opinion in an informal discussion, about using this treatment in our culture and whether any changes are required in the treatment. We will also ask you some questions to fill in about yourself.

**Privacy:** We assure you that your research information will be kept confidential. We will use ID numbers instead of your name. Your name will not be given to anyone without your consent. Only researchers will be able to look at your completed forms and all information will be password protected and any paperwork will be kept in a secure locked cabinet. It is possible that research papers may be published for scientific purposes; however neither your name nor that of any other participant will be used.

After the end of the 3 months, you will be invited to a meeting with one of the researchers to share your experience of using DIALOG+. This meeting will be recorded and the research assistant working for the team will transcribe the tape. When the tape is transcribed you will not be identified by name but by a code number. The tape will be kept securely. We will analyse the transcript on secure computers. Any comments that we quote in our final report will be anonymised.

**Risk /discomforts:** We believe that this study is safe and do not expect you to suffer any harm or injury because of taking part. However, if you become distressed whilst taking part, we will stop the session and ask if you want to continue. You will receive support from the clinical services for your distress, if required. If you want to continue, we will give you time to recover before continuing

**Benefits:** You may like to try the new DIALOG+ treatment in your mental health appointment as it may help improve the communication between you and the mental health professional you see in regular clinical meetings, and we hope that this will help improve your quality of life.

**Alternatives to procedures or treatment:** You have the choice of not being part of the study and continuing care as usual with your mental health professional. If you decide to withdraw from the study, the researchers will continue using the information you have provided to them up to that point, unless you state you do not want them to.

**What happens if you choose not to be part of the study?**

If you decide not to participate in this research, your treatment Karwan-e-Hayat/JPMC will not be impacted. Even after agreeing to participate in the study, you may stop participating at any time.

**Cost of taking part in research:** You do not have to pay for taking part in this study, we only require some of your time.

**Payment for taking part in research**: There is no payment for the treatment sessions you will recieve as part of the study as these will be your usual appointments with your mental health professional. However you will be paid PKR 850 for your meeting with the researcher at 3 months to discuss your experiences.

Do you agree to participate in this research study?

Please tick the ones that apply:

Do you agree to be part of this research study?

Do you agree to take part in the qualitative interview at the end of the 3 month study period?

Do agree to have the qualitative interview session audio recorded?

By signing the consent form it means that you give your consent to participate voluntarily in these sessions.

Patient agrees to participate in the study and I have given them an opportunity to answer queries***:***

Participant Name: __________________________________________________________

Participant Signature:_____________________________ Date: _________________

Sign of person taking consent: _____________________________ Date: _________________

You have consented to be part of this research study. If you have questions about this research, you may contact the Co-PI (name) from Monday to Friday 9 am to 5 pm at (phone number).

If you have questions about your rights in a research study as a volunteer, call or contact the IRB office between office hours on Monday to Friday 9am – 5pm at (phone number).
