# Supplementary material for: Exploring the feasibility and acceptability of DIALOG+ (a structured digital communication tool) in strengthening psychiatric care in India and Pakistan: a qualitative pilot study
Source: BMJ Open. 2025 Aug 12;15(8):e091852. doi: 10.1136/bmjopen-2024-091852 (PMC12352267; doi:10.1136/bmjopen-2024-091852)
Supplement: Supplementary file 2 [file bmjopen-2024-091852.reviewer_comments.pdf]

PEER REVIEW HISTORY

BMJ Open publishes all reviews undertaken for accepted manuscripts. Reviewers are asked to complete a checklist review form and are provided with free text boxes to elaborate on their assessment. These free text comments are reproduced below.

ARTICLE DETAILS

Title (Provisional)

Exploring the Feasibility and Acceptability of DIALOG+ (a Structured Digital Communication Tool) in Strengthening Psychiatric Care in India and Pakistan: a Qualitative Pilot Study

Authors

Qureshi, Onaiza; Divya, Kasthuri; Dawood, Mehwish; Davis, Syjo; Venkatraman, Lakshmi; Baig, Maria; Priya, Krishna; Peppl, Renata; Pari, Manikandan; Ramachandran, Padmavati; Pasha, Aneeta; Sajun, Sana Zehra Zehra; Sarwar, Hufsa; Shahab, Areeba; Bird, Victoria Jane

---

VERSION 1 - REVIEW

**Reviewer**	**1**
**Name**	**Allerby, Katarina**
**Affiliation**	**University of Gothenburg, Health and Care Sciences**
**Date**	**08-Nov-2024**
**COI**	**None**

I find this paper very valueble and overall sound. Congratulations on i nicely written paper in an important field or research!

I however have some comments which need attention concerning the matching of aim and study, description of "themes/main-codes" and handling of qualitative results along with some minor comment. Please find these in the added file where I used comments to hightlight my questions/comments/suggestions (the final ones are in the checklist at ther end!).

Good luck with revisions and again; well done with this valueble research project!

****** The reviewer provided a marked copy with additional comments. Please contact the publisher for full details. ******

Reviewer 2

Name Bhavsar, Vishal

Affiliation Institute of Psychiatry Department of Health Service and Population Research, Department of Health Services and Population Research

Date 05-Mar-2025

COI None

Thank you for this good paper.

Include a description of what DIALOG+ is in the title.

Replace “within” with “in” in the title, IMO.

Line 33- specify “ limited evidence” say it has not been evaluated in South Asia, if this is the case.

Line 35- include what the relation was of these participants to DIALOG+.

This is implied but can you state what the qual methods were trying to answer? I.e. say we carried out interviews to examine the feasibility and acceptability of the tool for routine clinical practice.

Introduction

Schizophrenia and bipolar don’t need capitals.

Significant rates of premature mortality- compared to what? Premature implies a reference point, so this needs specifying. Similarly, a longer duration of untreated illness compared to what?

I would recommend some expansion of what you mean by “biomedical in nature”, citing evidence.

Can you expand on “little is known” at line 75? If little is known about existing treatment, doesn’t that call for further knowledge on existing delivery(rather than new intervention per se)? Relatedly the justification provided at line 82 seems key, so should also be expanded to make clear why DIALOG was needed.

Line 91 doesn’t need to refer to the funder IMO as funding is presented elsewhere.

Lines 430-432 sound very interesting but I didn’t quite follow so could you expand?

Thank you for mention the LPC on psychoses in the global context- I wonder if this is context to your study, and if so would be better placed in the introduction?

At the discussion, I am reminded that I think in terms of feasibility you were primarily exploring feasibility of implementing DIALOG In clinical practice, and it might be worth specifying that in the method and intro, given feasibility can mean different things.

References are inconsistently formatted, please check/amend.

VERSION 1 - AUTHOR RESPONSE

Peer Review: 1
Dr. Katarina Allerby, University of Gothenburg, Sahlgrenska University Hospital Comments to the Author:
I find this paper very valueble and overall sound. Congratulations on i nicely written paper in an important field or research!
I however have some comments which need attention concerning the matching of aim and study, description of "themes/main-codes" and handling of qualitative results along with some minor comment. Please find these in the added file where I used comments to hightlight my questions/comments/suggestions (the final ones are in the checklist at ther end!).

1. I would present D+ as a person-centered conversational tool to strengthen patient participation in mental health care
    a. Authors: Thank you for your suggestion to strengthen the introduction to the abstract. This has now been changed to " DIALOG+ is a digital structured communication tool to strengthen the quality of person-centric psychiatric care by improving the therapeutic alliance between care providers and mental health service users . " in line 30
2. I did not understand what was meant by "the script" for IDIs...
    a. Authors: This is good feedback for us as script is not a well known word for topic guide. More detail on this is given in the methods so we have simplified this statement and removed reference to the word in the abstract.
3. I suggest cutting this part, I found it confusing as D+ does not really include this.
    a. Authors: We have removed this as you suggested
4. Is this a finding from your study? I would concentrate this section on implications of your findings!
    a. Authors: We appreciate your comment here. We believe that this statement is an implication as it connects our findings to broader practical applications of the identified gaps in future directions within the field of mental health
5. Later som BP ar en psykos sjukdom. (Sounds like BP is a psychotic disorder.)

    This is an important point - we have clarified to say bipolar disorder with psychotic feasutres in line 87

6. Ej under tonar val? (not during teenage years, right?)

    a. Authors: There is evidence that onset of psychotic disorders can begin in the teenager years with a concent zration in the early 20's - reference - https://www.sciencedirect.com/science/article/abs/pii/S0010440X08000862. We have added this reference in line 99 as well as the bibliography

7. Vad betyder detta? (what does this mean?)

    a. Authors: Thank you for highlighting that this isn't clear - we have included more detail: 'referring to the gap between the proportion of people who require care but do not receive it' in line 92

8. Kolla ref 9! (check reference 9!)

    a. Authors: We have checked and can confirm this is the right reference for the statements made as it discusses the complications of physical health outcomes for people with severe mental illnesses such as psychosis

9. on how big a (psychiatric) population?

    a. Authors: Thank you for bringing this up, unfortunately we do not have this data due to a lack of national level monitoring and evaluation

10. Kolla ref {19-21} (check reference 19-21)

    a. Authors: We have checked these and references 19-21 are all quoting papers on the evidence behind DIALOG in difference resource settings and their effectiveness in improving QoL and treatment outcomes.

11. I suggest rephrasing to clearify this is a qualitative study which serves to provvide insights into feasibility and acceptability. To assess feasibility a quantitative and randomized pilot or study would be needed.

    a. Authors: We appreciate the feedback and have clarified in-text that we wish to qualitatively assess feasibility and acceptability in the aims and objectives line 129-130

12. This aim does not seem to get an thorough answer in the paper, maybe cross and rewrite as some sort of implication of the study outcome. if you wish to keep as aim, it should be more clearly discussed or highlighted.

    a. Authors: We have removed this as you suggested

13. Im having difficulties in understanding why these individuals are included as they have no experience of the D+ tool (how can they answer questions on the use of D+ without any knowledge or experience?)? Could you elaborate on the rational for including them please?

This is a valid point and we have elaborated upon this section as recommended 'Since the overarching project aimed to scale up DIALOG+ implementation across additional mental health settings and clinicians, including these stakeholders was necessary in order to understand feasibility and acceptability perspectives into the broader mental health infrastructure, resources and context that would influence the adoption of DIALOG+. Prior to the interviews, these individuals were given an orientation to DIALOG+ protocol and clinical application so that they were familiar with its intended purpose and would be better able to respond to the questions.' lines 175-182

14. kolla ref 24 (check reference 24)

    a. Authors: I can confirm this is the correct reference and the guide was adapted from this research study pilot - https://www.cambridge.org/core/journals/bjpsych-international/article/feasibility-and-acceptability-of-a-solutionfocusedapproach-to-strengthen-lay-counselling-for-common-mental-disorders-dialogin-pakistan-mixed-methods-study/0493BFC18F6A9556F3F9AE9E32389BE7

15. Did you follow any described method of analysis for this qualitative analhysis? Reference? How did you handle personal bias? I miss something on how disagreements were settled, it seems to me as if 8 people sit down and code, you would get quite a lot of different codes, how did you narrow it down? Its ccommon to address reflexivity in

    a. Authors: Thank you for this important suggestion to improve the rigor of our methods section. We used an inductive approach to code and analyse the interviews using elements of thematic analysis to develop and find common patterns of findings across the database. We also used a directed approach and this hybrid model has also been used by Fereday and Muir-Cocraine (2006) to allow thematization to emerge organically while still allowing researchers to incorporate relevant programmatic objectives (i.e. feasibility and acceptability) when exploring participant inputs - lines 236-242
    b. Authors: How we included reflexivity and managed disagreements is outlined in lines 249-254.

16. The result is devided into two preset themes)acceptebility and feasibiliity) based on the aims, this should be described in the analysis section as I guess it was preset conditions that guided the analysis

    a. Authors: Yes that is correct, we have also clarified this in line 241 under data analysis

17. Could you elaborate something on how this was done and how input was considered/affected the analysis/result?

    a. Authors: While we shared our topic guide for the interviews with our Lived Experience Advisory Panel, but no revisions were suggested or ultimately made. This has been updated in our manuscript.

18. Table 3 (secondary care) could patients have their care at both secondary and tertiary care centers, (as this column exceeds 100%)?

Yes, the proportion is mixed as most participants attended multiple healthcare settings. This has been clarified in the table through as asterisks and lines 294-295

19. I am not familiar with this approach of having a deductive themeatization, is there any reference you could provide to guide the reader further on this approach?

    a. Authors: Yes, we have elaborated on the hybrid approach we took within the procedures i.e. 'analysis combined inductive thematic analysis with directed elements which has been showcased by Fereday and Muir-Cocraine (2006) to allow thematization to emerge organically while still allowing researchers to incorporate relevant programmatic objectives (i.e. feasibility and acceptability) when exploring participant inputs' in lines 236-242

20. In the table you state that Aesthetics is a "man code" not a theme. A theme is, to my knowledge, suppose to capture an abstract notion across the whole data set or a section of the data (i.e. across categories or what you refer to as "main codes"). Whatever you decide to table it, make sure to use the same lable throughout!

    a. Authors: Thank you for highlighting this error, we have corrected it to say ‘code’ and have standardised it throughout the results section

21. It seems to me that this is quite "thin" and unprocessed. Was this all that emerged as facilitators from clinicians?

    a. Authors: Yes but just within this particular code which did not emerge as a significant component of the app for most participants - there were more detailed facilitators under the other codes

22. theme?

    a. Authors: Corrected to code

23. One

    a. Authors: Corrected

24. Interesting point as in western care its often not doctors who use D+ but case managers, RNs or other mental health care workers.

    a. Authors: Yes that is a valid observation - In this DIALOG+ pilot there were more psychiatric professionals from both India and Pakistan

25. This is almost the same text as in quote below, I think you can allow for some abstraction and rephrasing to something like "clinicians highlighted its user-friendliness and proposed its usefullness also in less educated staff."

    a. Authors: Thank you for this suggestion to improve the narration of the results. We have now revised this and stardardised it across the results section

26. I react to the frequent referral to one statement or one participant (this goes for the entire result section)- the text should summarize the data and describe the central content, not exemplify some of it. I do believe thee result section will benefit from a review of this.

    a. Authors: This is well noted and appreciated - we have now revised and summarised the central findings under each main code across the results section.

27. theme?

    a. Authors: Corrected to code

28. In this section you deliver a good summary of the content in this "sub-code"! A good example of what I was wishing for in other sections of the result (as described in previous comment).

    a. Authors: Thank you for the feedback, we have now strengthened the results section as per this recommendation.

29. ? (question mark for "theme" word)

    a. Authors: Corrected

30. ? (question mark for "theme" word)

    a. Authors: Corrected

31. ? (question mark for "theme" word)

    a. Authors: Corrected

32. Very interesting and an important find in the study!

    a. Authors: Agreed - the importance of including family support must be carefully considered with respect to building the personal autonomy of the clients in their treatment decisions which DIALOG+ aims to strengthen.

33. Caution about generalizing qualitative findings generally. Findings cannot be seen to represent any population but serve as example of existing opinions and experiences within the population.

    a. Authors: We agree with you and have removed this statement as per your recommendation

34. again, the result cannot be seen as representing these large populations.

    a. Authors: Agreed and removed as per feedback

35. Page 7 - needs elaboration (This relates to item 7 on the COREQ checklist ‘What did the participants know about the researcher’)

    a. Authors: Thank you for highlighting this oversight - we have now added in this section within the procedures lines 220-224

36. Page 7 - not reported (This relates to item 9 on the COREQ checklist 'What methodological orientation was stated to underpin the study?')

    a. Authors: We have now added a more comprehensive description of the methodological orientation informing the analysis of the study under the data analysis section - 'the qualitative analysis undertook an inductive approach to code and analyse the IDIs and FGDs using elements of thematic analysis for the synthesis ' in lines 236-242.

37. N/A - How come this is not applicable in qualitative research (This relates to item 22 on the COREQ checklist 'Was data saturation discussed?')

    a. Authors: Thank you for bringing this the issue of data saturation as noted in our COREQ checklist. We have clarified this in the data analysis section under lines 242-245 to mention that 'data saturation was not a primary consideration in this qualitative analysis as our focus at this stage was to evaluate the feasibility and acceptability of DIALOG+ within the broader research project, with the goal of informing subsequent adaptations to optimize its adoption during wider implementation among psychiatric clinicians'

Reviewer: 2
Dr. Vishal Bhavsar, Institute of Psychiatry Department of Health Service and Population Research
Comments to the Author:
Thank you for this good paper.

1. Include a description of what DIALOG+ is in the title.

    a. Authors: As suggested we have included a short description of DIALOG+ in the title: Exploring the Feasibility and Acceptability of DIALOG+ (a Structured Digital Communication Tool) in Strengthening Psychiatric Care within India and Pakistan: a Qualitative Pilot Study

2. Replace "within" with "in" in the title, IMO.

    a. Authors: We have corrected this as recommended.

3. Line 33- specify " limited evidence" say it has not been evaluated in South Asia, if this is the case.

    a. Authors: Noted and clarified in the abstract to say 'DIALOG+ has been studied extensively in European and African contexts, but there is limited evidence for its evaluation in low-resource settings within South Asia' in lines 32-33

4. Line 35- include what the relation was of these participants to DIALOG+.

    a. Authors: This is well noted, we have now clarified in lines 33-36 to say 'This qualitative study conducted interviews to explore the experiences of participants recruited in the DIALOG+ pilot and wider stakeholder perspectives…'

5. This is implied but can you state what the qual methods were trying to answer? I.e. say we carried out interviews to examine the feasibility and acceptability of the tool for routine clinical practice.

   a. Authors: The reference to interviews has been included in the abstract on line 34

6. Introduction - Schizophrenia and bipolar don't need capitals.

   a. Authors: Noted and revised as per your suggestion

7. Significant rates of premature mortality- compared to what? Premature implies a reference point, so this needs specifying. Similarly, a longer duration of untreated illness compared to what?

   a. Authors: Thank you for highlighting this oversight, we have added 'relative to the general population' as outlined in the literature pertaining to this statement in lines 89-90.

8. I would recommend some expansion of what you mean by "biomedical in nature", citing evidence.

   a. Authors: We have elaborated on the statement by including additional information 'biomedical in nature (e.g. prioritizing pharmacological solutions for mental health concerns)' in line 100. The references included already point to this description.

9. Can you expand on "little is known" at line 75? If little is known about existing treatment, doesn't that call for further knowledge on existing delivery (rather than new intervention per se)? Relatedly the justification provided at line 82 seems key, so should also be expanded to make clear why DIALOG was needed.

   a. Authors: This is an important point – we have now included a segue to expand upon the justification for more resource effective and patient centred approaches like DIALOG on lines 110-112 right before it is officially introduced.

10. Line 91 doesn't need to refer to the funder IMO as funding is presented elsewhere.

   a. Authors: This is noted – however we wanted to introduce the wider program under which this pilot research study was nested to add context to the why we were exploring feasibility and acceptability of DIALOG+ - as a precursor to a more rigorous clinical evaluation.

11. Lines 430-432 sound very interesting but I didn't quite follow so could you expand?

   a. Authors: We appreciate this feedback for clarity and have further expanded on this point in lines 497-499.

12. Thank you for mention the LPC on psychoses in the global context- I wonder if this is context to your study, and if so would be better placed in the introduction?

   a. Authors: Thank you for this feedback, we intentionally positioned this point in the discussion to showcase our implications to contribute to the wider knowledge dissemination on psychosis care in global settings.

13. At the discussion, I am reminded that I think in terms of feasibility you were primarily exploring feasibility of implementing DIALOG In clinical practice, and it might be worth specifying that in the method and intro, given feasibility can mean different things.

a. Authors: We appreciate this feedback and also received it from the first reviewer so we have amended our aims section accordingly to make our research objective clearer in lines 129-131

14. References are inconsistently formatted, please check/amend.

a. Authors: Thank you for highlighting this - We have now revised and reincorporated the references section to accommodate the Vancouver style as per BMJ Open's requirements.

VERSION 2 - REVIEW

**Reviewer**	**1**
**Name**	**Allerby, Katarina**
**Affiliation**	**University of Gothenburg, Health and Care Sciences**
**Date**	**27-May-2025**
**COI**	

Thank you for a nicely revised manuscript. I find it easy to follow and understand and it adresses such important topics on how to make use of interventions in times and places of limited resources!

I have uploaded a file where I've made a few comments. It's mainly just grammar or short clearifications that are needed, so very small revision!

Again, congratulations on a very important piece of research and a nicely performed report on it!

**** **The reviewer provided a marked copy with additional comments. Please contact the publisher for full details.** ****

**Reviewer**	**2**
**Name**	**Bhavsar, Vishal**
**Affiliation**	**Institute of Psychiatry Department of Health Service and Population Research, Department of Health Services and Population Research**
**Date**	**09-May-2025**
**COI**	

I have nothing further to add.

VERSION 2 - AUTHOR RESPONSE

Reviewer: 1
Dr. Katarina Allerby, University of Gothenburg, Sahlgrenska University Hospital
Comments to the Author:
Thank you for a nicely revised manuscript. I find it easy to follow and understand and it adresses such important topics on how to make use of interventions in times and places of limited resources!

I have uploaded a file where I've made a few comments. It's mainly just grammar or short clearifications that are needed, so very small revision!

Again, congratulations on a very important piece of research and a nicely performed report on it!

Authors response: Thank you for your positive comments and your practical feedback on strengthening our manuscript.

Peer Review: 1

1. This sentence come off abit weird, maybe going back to the easier form "A hybrid inductive and thematic...."
    a. Authors: We have now reverted back to the original phrase as suggested.

2. Is Bipolar disorder really considered a psychotic disorder? To my knowledge it is considered an affective disorder.
    a. Authors: Yes you are right but we have mentioned it here given the common occurrence of psychotic symptoms in people with bipolar disorder e.g. delusions. We have now corrected the sentence to accurately reflect the difference in classification.
3. What was the opinions of the pilot participants? Maybe expanding abit on this part as this is an important and hard-to-change prerequisite for the clinicians.
    a. Authors: This was a commonly mentioned fact by the pilot clinicians and has not been bought up as such in the interviews with pilot patients. However, we have expanded upon this point as you rightly pointed out it is a key finding to the following: “One of the main experienced and anticipated challenges related to the time taken for DIALOG+ sessions in the context of busy and space-limited outpatient settings. This finding was reported by multiple clinicians in both countries, quoting high patient loads and limited individual time with patients as a predominant barrier to the adoption of DIALOG+ in routine clinical practice (as it required more targeted one-on-one support with each patient).”

The remainder of the comments specific to grammatical errors have also been addressed as per the feedback.

Reviewer: 2
Dr. Vishal Bhavsar, Institute of Psychiatry Department of Health Service and Population Research
Comments to the Author:

I have nothing further to add.

Author response: We thank the peer reviewer for their valuable feedback lending towards the strengthening of our manuscript.
